# Is Health Insurance Literacy Associated With Financial Hardship Among Cancer Survivors? Findings From a National Sample in the United States

**DOI:** 10.1093/jncics/pkz061

**Published:** 2019-10-21

**Authors:** Jingxuan Zhao, Xuesong Han, Zhiyuan Zheng, Matthew P Banegas, Donatus U Ekwueme, K Robin Yabroff

**Affiliations:** 1 Surveillance and Health Services Research Program, American Cancer Society, Atlanta, GA; 2 The Center for Health Research, Kaiser Permanente, Portland, OR; 3 Division of Cancer Prevention and Control, National Center for Chronic Disease Prevention and Health Promotion, Centers for Disease Control and Prevention, Atlanta, GA

## Abstract

Little is known about the association between health insurance literacy and financial hardship among cancer survivors. Using the 2016 Medical Expenditure Panel Survey Experiences with Cancer self-administered questionnaire, we evaluated the associations between health insurance literacy and medical financial hardship and nonmedical financial sacrifices among adult cancer survivors in the United States. Of the survivors, 18.9% aged 18–64 years and 14.6% aged 65 years and older reported health insurance literacy problems. In both age groups (18–64 and ≥65 years), from multivariable logistic regressions, survivors with health insurance literacy problems were more likely to report any material (adjusted odds ratio [AOR] = 3.02, 95% confidence interval [CI] = 1.53 to 5.96; AOR = 3.33, 95% CI = 1.69 to 6.57, respectively) or psychological (AOR = 5.53, 95% CI = 2.35 to 13.01; AOR = 8.79, 95% CI = 4.55 to 16.97, respectively) hardship, as well as all types of nonmedical financial sacrifices than those without these problems. Future longitudinal studies are warranted to test causality and assess whether improving health insurance literacy can mitigate financial hardship.

Rising costs of cancer care result in financial hardship for cancer survivors, even among those with health insurance (1). Growing evidence indicates that many adults have limited knowledge, ability, and confidence to obtain, evaluate, and use health insurance information (health insurance literacy), which may prevent optimal use of health plan benefits and lead to unnecessary medical spending (2). Therefore, health insurance literacy has been proposed as an important, potentially modifiable patient characteristic for preventing financial hardship (3). With growing numbers of newly insured individuals following implementation of the Affordable Care Act, the improvement of health insurance literacy is receiving increasing attention (4,5). To date, however, little is known about the associations between health insurance literacy and medical financial hardship and nonmedical financial sacrifice in cancer survivors.

We identified adult cancer survivors from the 2016 Medical Expenditure Panel Survey (MEPS) Experiences with Cancer self-administered questionnaire CSAQ, a nationally representative household survey (6) addressing financial hardship, health insurance coverage, and access to care related to cancer, its treatment, and lasting effects of treatment. The response rate was 46.0% for the 2016 core MEPS and 81.2% for the cancer self-administered questionnaire among MEPS respondents, resulting in an overall response rate of 37.4% (6). Detailed information on the MEPS is presented in eMethods (available online).

Health insurance literacy was measured by the question, “Did you ever have a problem understanding health insurance or medical bills related to your cancer, its treatment, or the lasting effects of that treatment?” (yes or no). Consistent with earlier studies (1,7), medical financial hardship was categorized into three domains: 1) material (eg, problems paying medical bills); 2) psychological (eg, worry about paying medical bills); and 3) behavioral (eg, delaying or forgoing care because of cost). Nonmedical financial sacrifices were measured by changes in spending, living situation, or use of savings. Exact wording of questions is listed in Supplementary Table 1 (available online).

Descriptive statistics were used to characterize the sample. We used multivariable logistic regressions to separately evaluate the associations between health insurance literacy and medical financial hardship and nonmedical financial sacrifices, adjusted for potential confounders. Analyses were stratified by age group (18–64 years and ≥65 years) by including an interaction term of age group by health insurance literacy in models, because of differences in insurance coverage and financial hardship (1). We also conducted sensitivity analysis to assess whether the associations differ by some important risk factors of financial hardship, including health insurance, family income, time since last cancer treatment, and number of conditions (Supplementary Tables 2–9, available online). Both descriptive statistics and multivariable logistic regressions accounted for the complex survey design and nonresponse using SUDAAN (9). Statistical tests were two-sided, and results were considered significant if *P* was less than .05. This study was exempted from institutional review board approval because all study data were deidentified and publicly available.

The majority of cancer survivors were aged 65 years and older (Table 1). In both age groups, most survivors were female, non-Hispanic white, married, and with private health insurance. Compared with survivors aged 18–64 years, those aged 65 years and older were less likely to have attended college and reported more health conditions (both *P* < .01). Of the survivors, 18.9% (18–64 years) and 14.6% (≥65 years) reported having problems understanding health insurance or medical bills. Survivors aged 18–64 years with only public insurance or uninsured (*P* = .011), with family income no more than 400% of the federal poverty line (*P* = .027), and less than 5 years since last cancer treatment (*P*= .004) were more likely to report health insurance literacy problems (Supplementary Figure 1, available online).

**Table 1.** Characteristics of cancer survivors, Medical Expenditure Panel Survey Cancer Questionnaire, 2016 (n = 914)

Characteristics	18–64 y			≥ 65 y			χ^2^*P*
	No.	Weighted %*	χ^2^*P*	No.	Weighted %*	χ^2^*P*	
Total cancer survivors†	389	100.0		525	100.0		—
Age group, y			0.995			.546	—
18–54	203	50.0		—	—		
55–64	186	50.0		—	—		
65–74	—	—		255	48.6		
75– ≥85‡	—	—		270	51.4		
Sex			<.001			<.001	.008
Female	276	68.1		297	57.2		
Male	113	31.9		228	42.8		
Race/ethnicity			<.001			<.001	.042
Non-Hispanic white only	233	78.0		380	83.6		
All other races/ethnicities	156	22.0		145	16.4		
Current marital status			<.001			.132	.009
Married	235	64.4		259	53.7		
Not married	154	35.6		266	46.3		
Education§			<.001			<.001	.002
High school graduate or less/missing	173	35.8		269	48.6		
Some college or more	216	64.2		256	51.4		
Current family income as percentage of federal poverty line			<.001			.528	.114
Low income ≤138%	109	20.2		127	19.1		
Middle income 139–400%	137	30.2		207	38.0		
High income >400%	143	49.6		191	42.9		
Current health insurance for age 18–64 y∥			<.001			—	—
Age 18–64, any private	251	74.9		—	—		
Age 18–64, public only or uninsured¶	138	25.1		—	—		
Current health insurance for age ≥ 65 y∥			—			<.001	—
Age ≥65, Medicare and private*	—	—		270	56.5		
Age ≥65, Medicare and other public*	—	—		79	11.1		
Age ≥65, Medicare only*	—	—		176	32.4		
Number of current conditions, excluding cancer#	—	—	0.735			<.001	<.001
0–1	187	49.2		84	14.2		
2–8	202	50.8		441	85.8		
Years since last cancer treatment§			0.039			<.001	.282
<5	188	44.8		210	40.6		
≥5 or never treated/missing	201	55.2		315	59.4		
Ever had problem understanding health insurance or medical bills			<.001			<.001	.196
Yes	83	18.9		79	14.6		
No	306	81.1		446	85.4		

* Weighted percentages were calculated based on the Medical Expenditure Panel Survey (MEPS) design and sample weight. The SUDAAN “subpopn” command was used to form the analytic sample and keep the weight information.

† Adult cancer survivors were defined as those aged 18 years or older reported ever being told by a doctor or other health professional that they had cancer or a malignancy of any kind. Individuals diagnosed only with nonmelanoma skin cancer and/or skin cancer with unknown kind (n = 267), aged 65 years or older without Medicare coverage (n = 6), or who did not respond to the health insurance and financial literacy question (n = 49) were excluded from this study.

‡ Age top-coded 85 years or older by the MEPS.

§ Consistent with earlier studies (8), cancer survivors with missing information on educational attainment (<0.5%) were combined with high school graduate or less; cancer survivors who did not respond to the time since last cancer treatment question (<1%) were combined with the more than 5 years or never treated group.

∥ Public insurance included Medicare, Medicaid, State Children’s Health Insurance Program, and/or other public hospital and physician coverage. TRICARE/CHAMPVA was treated as private coverage, as were employer-based, union-based, and other private insurance.

¶ For age 18–64 years, uninsured adults were also included because they were likely to be covered in the past and respond to the health insurance literacy question based on earlier experience. Public only or uninsured were combined because of the small sample size for the uninsured (n = 20).

# Conditions include arthritis, asthma, diabetes, emphysema, heart disease (angina, coronary heart disease, heart attack, other heart condition and/or disease), high cholesterol, hypertension, and stroke. Consistent with earlier studies (8), cancer survivors with missing information on angina history (<0.5%) were categorized as no angina history.

In both age groups (18–64 and ≥65 years), health insurance literacy problems were associated with higher likelihood of reporting any material (odds ratio [OR] = 3.02, 95% confidence interval [CI] = 1.53 to 5.96; OR = 3.33, 95% CI = 1.69 to 6.57; respectively) or psychological (OR = 5.53, 95% CI = 2.35 to 13.01; OR = 8.79, 95% CI = 4.55 to 16.97; respectively) hardship (Table 2). We observed a higher likelihood of reporting all measures of financial sacrifices among those who had health insurance literacy problems in both age groups (all *P* < .05). Compared with survivors aged 65 years and older, a stronger association between health insurance literacy and financial sacrifices were observed among those aged 18–64 years (*P* = .010). Among survivors aged 65 years and older, the associations between health insurance literacy and material and psychological financial hardship were stronger among survivors with at least 5 years since last cancer treatment than those within 5 years of treatment (Supplementary Table 7, available online).

**Table 2.** Associations of health insurance literacy problems and financial hardship and financial sacrifices among cancer survivors, by age group, Medical Expenditure Panel Survey Cancer Questionnaire, 2016 (n = 914)

Characteristics	18–64 y	≥ 65 y*	Wald F *P*‡
	AOR (95% CI)†	AOR (95% CI)†	
Material financial hardship§			
Had to borrow money or go into debt	4.04 (2.07 to 7.88)	2.51 (0.97 to 6.50)	.414
Unable to cover share of the costs of medical care	1.81 (0.89 to 3.67)	3.15 (1.49 to 6.64)	.263
Any material financial hardship‖	3.02 (1.53 to 5.96)	3.33 (1.69 to 6.57)	.836
Psychological financial hardship§			
Worried about paying large medical bills	4.79 (2.32 to 9.89)	8.85 (4.66 to 16.82)	.174
Worried about family’s financial stability	6.36 (3.00 to 13.48)	9.35 (4.78 to 18.30)	.443
Concerned about keeping job and income or earnings	4.56 (2.45 to 8.48)	3.71 (1.76 to 7.83)	.669
Any psychological financial hardship¶	5.53 (2.35 to 13.01)	8.79 (4.55 to 16.97)	.372
Behavioral financial hardship§			
Delay or forgo cancer care because of cost of			
Prescription medicine	3.70 (1.45 to 9.48)	3.44 (1.38 to 8.60)	.910
Visit to specialist	2.22 (0.82 to 5.98)	3.59 (0.90 to 14.31)	.586
Follow-up care	1.71 (0.80 to 3.64)	2.92 (1.12 to 7.64)	.378
Any behavioral financial hardship#	1.96 (0.96 to 4.02)	1.09 (0.57 to 2.09)	.209
Nonmedical financial sacrifices§			
Reduce spending on vacation or leisure activities	3.57 (1.78 to 7.16)	2.51 (1.51 to 4.17)	.421
Delay large purchases (eg, car)	3.98 (1.95 to 8.13)	3.18 (1.55 to 6.55)	.657
Reduce spending on basics (eg, food and clothing)	4.59 (2.32 to 9.09)	2.71 (1.40 to 5.24)	.240
Use savings set aside for other purposes (eg, retirement, educational funds, family support)	6.89 (3.19 to 14.90)	5.03 (2.54 to 9.94)	.532
Make a change to living situation (eg, sold, refinanced, or moved to a smaller residence)	3.04 (1.27 to 7.29)	3.68 (1.64 to 8.24)	.748
Any financial sacrifices**	9.90 (3.77 to 26.00)	2.12 (1.20 to 3.75)	.010

* Age top-coded 85 years or older by the Medical Expenditure Panel Survey. AOR = adjusted odds ratio; CI = confidence interval.

† Reference group was cancer survivors who had no problems understanding health insurance or medical bills. Multivariable models adjusted for age group, sex, race and ethnicity, educational attainment, years since last cancer treatment, and current marital status, family income, health insurance coverage, and number of conditions. The SUDAAN “subpopn” command was used to form the analytic sample and keep the weight information.

‡ Wald F *P* tested the differences in the associations between health insurance literacy and financial hardship by age group (18–64 years, ≥65 years).

§ To keep sample size consistent with earlier studies, cancer survivors who did not respond to the financial hardship and financial sacrifices question were combined with those who responded that they had no hardship or sacrifices. The weighted nonresponse rates for the material and psychological financial hardship questions were between 1% and 3%. We were not able to calculate the nonresponse rates for the behavioral financial hardship and financial sacrifices questions because of the question design. More specifically, cancer survivors were asked to “mark all that apply” for behavioral financial hardship and financial sacrifices questions, which made it impossible to distinguish those who had no hardship or sacrifices (and did not mark any of the responses) from those who did not answer the question. We did not conduct a sensitivity analysis including only those who answered the question because they reported at least one term of hardship of sacrifices, which could make it a biased sample. However, the weighted percentages of reporting those items were comparable to other national representative estimates (8).

∥ Any material financial hardship was defined as having responded yes to one or more of the individual material financial hardship measures, including ever having to borrow money or go into debt because of cancer, being unable to cover share of the cost of medical care visits for cancer, and/or file for bankruptcy because of cancer. Filing for bankruptcy because of cancer was not shown as an individual measure because of the small number of observations and wide confidence interval.

¶ Any psychological financial hardship was defined as having responded yes to one or more of the individual psychological financial hardship measures, including ever being worried about paying large medical bills, worried about family’s financial stability, and/or concerned about keeping job and income or earnings because of cancer.

# Any behavioral financial hardship was defined as having responded yes to one or more of the adherence measures, including ever delaying, forgoing, or changing because of cost prescription medicine, visit to specialist, follow-up care, treatment, mental health services, and/or other. Treatment and mental health services were not shown as individual measures because of the small number of observations and wide confidence intervals.

** Any financial sacrifices were defined as having responded yes to one or more of the individual financial sacrifice measures, including reduce spending on vacation or leisure activities, delay large purchases, change basic spending, such as food and clothing, use savings set aside for other purposes, change living situation, and/or other sacrifice because of cancer. Other sacrifice was not shown as an individual measure because of the small number of observations and wide confidence interval.

In this study, we found health insurance literacy problems were associated with multiple domains of medical financial hardship and nonmedical financial sacrifices among cancer survivors in the United States. With accumulating evidence about the adverse effects of medical financial hardship (10) and rising costs of cancer care (11), financial burden of cancer is receiving increasing attention. As a potentially modifiable patient characteristic, health insurance literacy may be an important intervention lever for addressing financial problems.

Growing evidence suggests that health insurance literacy is a nationwide problem in the United States (12,13) and is associated with adverse effects (2,14,15). An earlier study found that Medicare beneficiaries who were not aware of the Part D donut hole (coverage gap) were more likely to report material and behavioral hardships (14). This study suggested that health insurance literacy problems could cause financial hardship by limiting the ability to avoid higher drug costs and navigate options in the health system. However, no specific interventions have been developed to address health insurance literacy so far. Interventions such as financial and health insurance navigation, decision aids, and more user-friendly and easier-to-read medical bills (13,16), which improve patients' understanding of health insurance and medical costs, could potentially be applied to improve health insurance literacy and benefit cancer survivors.

Our study is limited by the relatively small sample size, low response rate, and cross-sectional design. We were not able to evaluate other aspects of health insurance literacy, including information seeking, documentation, and self-efficacy (17,18), because of the unavailability of these data. We were also not able to control for cancer treatment or stage at diagnosis because of lack of data. We may have also underestimated the prevalence of health insurance literacy with self-reported data because of the gap in survivors’ self-estimates and their actual knowledge (19). Despite these limitations, we used national data to quantify the associations between health insurance literacy and financial hardship and financial sacrifices among cancer survivors. Future longitudinal studies are warranted to assess whether improving health insurance literacy can mitigate financial hardship.

## Notes

Affiliations of authors: Surveillance and Health Services Research Program, American Cancer Society, Atlanta, GA (JZ, XH, ZZ, KRY); The Center for Health Research, Kaiser Permanente, Portland, OR (MPB); Division of Cancer Prevention and Control, National Center for Chronic Disease Prevention and Health Promotion, Centers for Disease Control and Prevention, Atlanta, GA (DUE).

The findings and conclusions in this report are those of the authors and do not necessarily represent the official position of the Centers for Disease Control and Prevention. Dr Matthew P. Banegas has received research grants from AstraZeneca for projects outside the scope this research project. The other authors have nothing to declare.

## Supplementary Material

pkz061_Supplementary_Data
