# Supplementary material for: Is Health Insurance Literacy Associated With Financial Hardship Among Cancer Survivors? Findings From a National Sample in the United States
Source: JNCI Cancer Spectr. 2019 Oct 21;3(4):pkz061. doi: 10.1093/jncics/pkz061 (PMC7050003; doi:10.1093/jncics/pkz061)
Supplement: pkz061_Supplementary_Data [file pkz061_supplementary_data.docx]

**eTable 1**. Measures of medical financial hardship, financial sacrifice, and access to care among cancer survivors

| Measures | | MEPS questions |
| --- | --- | --- |
| Medical financial hardship | | |
| Material financial hardship measures | Borrow money or go into debt | Have you or has anyone in your family had to borrow money or go into debt because of your cancer, its treatment, or the lasting effects of that treatment? |
|  | Unable to cover share of the cost of cancer visits | Please think about medical care visits for cancer, its treatment, or the lasting effects of that treatment. Have you ever been unable to cover your share of the cost of those visits? |
|  | File for bankruptcy | Did you or your family ever file for bankruptcy because of your cancer, its treatment, or the lasting effects of that treatment? |
| Psychological financial hardship measures | Worried about having to pay large medical bills | Have you ever worried about having to pay large medical bills related to your cancer? |
|  | Worried about family’s financial stability | Have you ever worried about your family’s financial stability because of your cancer, its treatment or lasting effects of that treatment? |
|  | Concerned about keeping your job and income | Have you ever been concerned about keeping your job and income, or that your earnings will be limited in the future because of your cancer? |
| Behavioral financial hardship measures | Delay, forego, or have to make other changes because of cost | Did you ever delay, forego, or have to make other changes to any of the following cancer care because of cost? --Prescription medicine --Visit to specialist --Treatment (other than prescription medicine) --Follow up care --Mental health services --Other |
| Financial sacrifices | | |
| Financial sacrifices | Make any kinds of financial sacrifices because of cancer | Have you or your family had to make any other kinds of financial sacrifices because of your cancer, its treatment, or the lasting effects of that treatment? --Reduced spending on vacation or leisure activities --Delayed large purchases (e.g., car) --Reduced spending on basics (e.g., food and clothing) --Used savings set aside for other purposes (e.g., retirement, educational funds, family support) --Made a change to living situation (e.g., sold, refinanced, or moved to a smaller residence) --Other |
| Access to necessary care | | |
| Access to necessary care | Access to necessary care | At any time since you were first diagnosed with cancer, did you get all of the medical care, tests, or treatments that you or your doctor believed were necessary? |

Note: The time frame for all questions were from time since first diagnosed with cancer.

**eTable 2**. Associations of Health Insurance Literacy Problems and Financial Hardship and Financial Sacrifices among Cancer Survivors Aged 18-64 Years, by Health Insurance Coverage, Medical Expenditure Panel Survey Cancer Questionnaire, 2016 (N=389)

|  | Any private | Public only and uninsured | Wald F *P* ^†^ |
| --- | --- | --- | --- |
|  | Adjusted OR and 95% Confidence Intervals* | Adjusted OR and 95% Confidence Intervals* |  |
| Material financial hardship |  |  |  |
| Had to borrow money or go into debt | 2.77 (1.16 - 6.61) | 6.68 (1.93 - 23.14) | 0.289 |
| Unable to cover share of the costs of medical care | 1.73 (0.61 - 4.92) | 1.94 (0.79 - 4.74) | 0.873 |
| Any material financial hardship ^‡^ | 3.09 (1.31 - 7.29) | 2.98 (1.15 - 7.71) | 0.952 |
| Psychological financial hardship |  |  |  |
| Worried about paying large medical bills | 5.72 (2.32 - 14.10) | 3.23 (1.25 - 8.33) | 0.390 |
| Worried about family's financial stability | 8.06 (2.95 - 22.02) | 4.20 (1.50 - 11.75) | 0.360 |
| Concerned about keeping job and income or earnings | 4.82 (2.11 - 11.02) | 3.53 (1.38 - 9.03) | 0.626 |
| Any psychological financial hardship ^§^ | 7.39 (2.38 - 22.91) | 3.14 (1.15 - 8.56) | 0.265 |
| Behavioral financial hardship |  |  |  |
| Delay or forgo cancer care because of cost -- |  |  |  |
| Prescription medicine | 3.59 (1.01 - 12.70) | 3.12 (0.97 - 10.00) | 0.862 |
| Visit to specialist | 3.01 (0.89 - 10.12) | 1.87 (0.56 - 6.18) | 0.535 |
| Follow up care | 1.98 (0.59 - 16.67) | 1.85 (0.60 - 15.69) | 0.935 |
| Any behavioral financial hardship ^\|\|^ | 1.86 (0.75 - 4.60) | 2.53 (0.81 - 7.93) | 0.647 |
| Financial sacrifices |  |  |  |
| Reduce spending on vacation or leisure activities | 4.89 (1.93 - 12.44) | 1.75 (0.70 - 4.36) | 0.122 |
| Delay large purchases | 4.56 (1.82 - 11.47) | 2.63 (0.98 - 7.08) | 0.402 |
| Basic spending | 5.96 (2.23 - 15.92) | 2.39 (0.98 - 5.86) | 0.175 |
| Savings set aside for other purposes | 10.71 (4.16 - 27.57) | 3.33 (1.02 - 10.87) | 0.128 |
| Living situation | 1.00 (0.26 - 3.79) | 6.03 (2.04 - 17.79) | 0.056 |
| Any financial sacrifices ^¶^ | 62.39 (7.73 - 503.66) | 2.72 (0.92 - 8.03) | 0.051 |

| * OR, odds ratio. Reference group was cancer survivors had no problems understanding health insurance or medical bills. Multivariable models adjusted for age group, sex, race/ethnicity, current marital status, educational attainment, current family income, years since last cancer treatment, and number of conditions. |
| --- |
| ^†^ Wald F *P* tested the differences in the associations between health insurance literacy and financial hardship across current health insurance coverage type. |
| ^‡^Any material financial hardship was defined as having responded yes to one or more of the individual material financial hardship measures, including ever having to borrow money or go into debt because of cancer, be unable to cover share of the cost of medical care visits for cancer, and/or file for bankruptcy because of cancer. File for bankruptcy because of cancer was not shown as an individual measure because of the small number of observations and wide confidence interval. |
| ^§^ Any psychological financial hardship was defined as having responded yes to one or more of the individual psychological financial hardship measures, including ever being worried about paying large medical bills, worried about family's financial stability, and/or concerned about keeping job and income or earnings because of cancer. |
| ^\|\|^ Any behavioral financial hardship was defined as having responded yes to one or more of the adherence measures, including ever delaying, forgoing, or changing because of cost prescription medicine, visit to specialist, follow up care, treatment, mental health services, and/or other. Treatment and mental health services were not shown as individual measures because of small number of observations and the wide confidence intervals.  ^¶^ Any financial sacrifices were defined as having responded yes to one or more of the individual financial sacrifice measures, including reduce spending on vacation or leisure activities, delay large purchases, change basic spending, such as food and clothing, use savings set aside for other purposes, change living situation, and/or other sacrifice because of cancer. Other sacrifice was not shown as an individual measure because of the small number of observations and wide confidence interval. |

**eTable 3**. Associations of Health Insurance Literacy Problems and Financial Hardship and Financial Sacrifices among Cancer Survivors Aged ≥65 Years, by Health Insurance Coverage, Medical Expenditure Panel Survey Cancer Questionnaire, 2016 (N=525)

|  | Medicare and private | Medicare and other public | Medicare only | Wald F *P* ^†^ |
| --- | --- | --- | --- | --- |
|  | Adjusted OR and 95% Confidence Intervals * | Adjusted OR and 95% Confidence Intervals * | Adjusted OR and 95% Confidence Intervals * |  |
| Material financial hardship |  |  |  |  |
| Had to borrow money or go into debt | 3.42 (0.73 - 16.03) | -- | 0.56 (0.10 - 3.19) | 0.072 |
| Unable to cover share of the costs of medical care | 2.79 (0.80 - 9.71) | 5.88 (0.85 - 40.85) | 2.16 (0.63 - 7.43) | 0.660 |
| Any material financial hardship ^‡^ | 2.83 (0.95 - 8.41) | 11.23 (1.64 - 76.85) | 2.11 (0.69 - 6.49) | 0.300 |
| Psychological financial hardship |  |  |  |  |
| Worried about paying large medical bills | 12.92 (4.79 - 4.86) | 19.36 (3.41 - 109.86) | 3.54 (1.44 - 8.71) | 0.105 |
| Worried about family's financial stability | 7.92 (3.07 - 20.40) | 18.65 (3.74 - 93.01) | 7.19 (2.43 - 21.25) | 0.580 |
| Concerned about keeping job and income or earnings | 6.02 (2.29 - 15.85) | 3.80 (0.61 - 23.62) | 1.55 (0.42 - 5.76) | 0.288 |
| Any psychological financial hardship ^§^ | 11.69 (4.41 - 30.94) | 17.26 (3.16 - 94.36) | 3.93 (1.43 - 10.74) | 0.221 |
| Behavioral financial hardship |  |  |  |  |
| Delay or forgo cancer care because of cost -- |  |  |  |  |
| Prescription medicine | 4.80 (1.29 - 17.84) | 5.57 (0.51 - 60.53) | 1.63 (0.32 - 8.39) | 0.518 |
| Visit to specialist | 2.84 (0.26 - 31.57) | -- | 3.72 (0.59 - 23.39) | 0.325 |
| Follow up care | 3.52 (0.67 - 18.50) | 0.98 (0.06 - 15.80) | 2.01 (0.47 - 8.54) | 0.733 |
| Any behavioral financial hardship ^\|\|^ | 0.93 (0.35 - 2.47) | 3.01 (0.69 - 13.08) | 0.85 (0.30 - 2.44) | 0.328 |
| Financial sacrifice |  |  |  |  |
| Reduce spending on vacation or leisure activities | 1.70 (0.71 - 4.04) | 18.19 (2.43 - 136.46) | 1.96 (0.75 - 5.11) | 0.093 |
| Delay large purchases | 3.00 (0.90 - 9.96) | 5.99 (0.90 - 39.98) | 2.00 (0.53 - 7.66) | 0.646 |
| Basic spending | 2.03 (0.52 - 7.99) | 18.92 (4.12 - 86.79) | 1.52 (0.43 - 5.40) | 0.054 |
| Savings set aside for other purposes | 5.11 (1.77 - 14.73) | 10.93 (2.44 - 49.00) | 2.70 (0.73 - 10.01) | 0.368 |
| Living situation | 1.68 (0.41 - 6.87) | -- | 5.04 (1.17 - 21.67) | 0.235 |
| Any financial sacrifices ^¶^ | 0.99 (0.44 - 2.21) | 4.62 (1.14 - 18.66) | 4.64 (1.73 - 12.45) | 0.058 |

| * OR, odds ratio. Reference group was cancer survivors had no problems understanding health insurance or medical bills. Multivariable models adjusted for age group, sex, race/ethnicity, current marital status, educational attainment, current family income, years since last cancer treatment, and number of conditions. |
| --- |
| ^†^ Wald F *P* tested the differences in the associations between health insurance literacy and financial hardship across current health insurance coverage type. |
| ^‡^Any material financial hardship was defined as having responded yes to one or more of the individual material financial hardship measures, including ever having to borrow money or go into debt because of cancer, being unable to cover share of the cost of medical care visits for cancer, and/or file for bankruptcy because of cancer. File for bankruptcy because of cancer was not shown as an individual measure because of the small number of observations and wide confidence interval. |
| ^§^ Any psychological financial hardship was defined as having responded yes to one or more of the individual psychological financial hardship measures, including ever being worried about paying large medical bills, worried about family's financial stability, and/or concerned about keeping job and income or earnings because of cancer. |
| ^\|\|^ Any behavioral financial hardship was defined as having responded yes to one or more of the adherence measures, including ever delaying, forgoing, or changing because of cost prescription medicine, visit to specialist, follow up care, treatment, mental health services, and/or other. Treatment and mental health services were not shown as individual measures because of the small number of observations and wide confidence intervals.  ^¶^ Any financial sacrifices were defined as having responded yes to one or more of the individual financial sacrifice measures, including ever reduce spending on vacation or leisure activities, delay large purchases, reduce basic spending, such as food and clothing, use savings set aside for other purposes, change living situation, and/or other sacrifice because of cancer. Other sacrifice was not shown as an individual measure because of the small number of observations and wide confidence interval. |

**eTable 4**. Associations of Health Insurance Literacy Problems and Financial Hardship and Financial Sacrifices among Cancer Survivors Aged 18-64 Years, by Federal Poverty Line, Medical Expenditure Panel Survey Cancer Questionnaire, 2016 (N=389)

|  | Low-income ≤138% federal poverty line | Middle-income 139-400% federal poverty line | High-income >400% federal poverty line | Wald F P ^†^ |
| --- | --- | --- | --- | --- |
|  |  |  |  |  |
|  |  |  |  |  |
|  | Adjusted OR and 95% Confidence Intervals * | Adjusted OR and 95% Confidence Intervals * | Adjusted OR and 95% Confidence Intervals * |  |
| Material financial hardship |  |  |  |  |
| Had to borrow money or go into debt | 5.14 (1.30 - 20.25) | 5.22 (1.90 - 14.33) | 1.66 (0.41 - 6.68) | 0.359 |
| Unable to cover share of the costs of medical care | 1.85 (0.55 - 6.14) | 1.30 (0.57 - 2.97) | 3.09 (0.84 - 11.39) | 0.531 |
| Any material financial hardship ^‡^ | 3.48 (1.13 - 10.75) | 2.65 (1.02 - 6.91) | 3.40 (1.03 - 11.21) | 0.924 |
| Psychological financial hardship |  |  |  |  |
| Worried about paying large medical bills | 1.93 (0.65 - 5.74) | 3.39 (1.23 - 9.31) | 4.85 (4.09 - 53.88) | 0.074 |
| Worried about family's financial stability | 3.36 (0.98 - 11.54) | 3.73 (1.41 - 9.85) | 23.01 (4.60 - 115.05) | 0.125 |
| Concerned about keeping job and income or earnings | 2.54 (0.84 - 7.67) | 4.90 (1.89 - 12.73) | 5.42 (1.56 - 18.85) | 0.564 |
| Any psychological financial hardship ^§^ | 2.53 (0.79 - 8.07) | 3.39 (1.07 - 10.75) | 27.96 (4.70 - 166.41) | 0.070 |
| Behavioral financial hardship |  |  |  |  |
| Delay or forgo cancer care because of cost -- |  |  |  |  |
| Prescription medicine | 3.14 (0.61 - 16.26) | 3.64 (0.89 - 14.87) | 3.65 (0.67 - 19.81) | 0.987 |
| Visit to specialist | 0.88 (0.15 - 5.04) | 9.82 (2.32 - 41.62) | 2.41 (0.46 - 12.58) | 0.066 |
| Follow up care | 2.13 (0.59 - 7.77) | 1.54 (0.42 - 5.67) | 2.35 (0.46 - 11.93) | 0.903 |
| Any behavioral financial hardship ^\|\|^ | 1.01 (0.31 - 3.35) | 3.02 (1.01 - 9.05) | 2.63 (0.83 - 8.33) | 0.271 |
| Financial sacrifice |  |  |  |  |
| Reduce spending on vacation or leisure activities | 1.84 (0.55 - 6.19) | 2.64 (0.83 - 8.42) | 7.00 (2.30 - 21.26) | 0.254 |
| Delay large purchases | 2.89 (0.84 - 9.89) | 4.43 (1.40 - 13.98) | 3.88 (1.20 - 12.60) | 0.864 |
| Basic spending | 4.89 (1.57 - 15.27) | 4.62 (1.37 - 15.63) | 3.69 (1.08 - 12.60) | 0.942 |
| Savings set aside for other purposes | 2.96 (0.73 - 12.02) | 5.89 (1.81 - 19.18) | 5.76 (4.90 - 50.69) | 0.127 |
| Living situation | 3.74 (1.00 - 13.93) | 2.92 (0.74 - 11.56) | 1.37 (0.13 - 14.08) | 0.734 |
| Any financial sacrifices ^¶^ | 2.09 (0.66 - 6.63) | 1.37 (3.03 - 50.90) | -- | <0.001 |

| * OR, odds ratio. Reference group was cancer survivors had no problems understanding health insurance or medical bills. Multivariable models adjusted for age group, sex, race/ethnicity, current marital status, educational attainment, current health insurance coverage, years since last cancer treatment, and number of conditions. |
| --- |
| ^†^ Wald F *P* tested the differences in the associations between health insurance literacy and financial hardship across current health insurance coverage type. |
| ^‡^Any material financial hardship was defined as having responded yes to one or more of the individual material financial hardship measures, including ever having to borrow money or go into debt because of cancer, being unable to cover share of the cost of medical care visits for cancer, and/or file for bankruptcy because of cancer. File for bankruptcy because of cancer was not shown as an individual measure because of the small number of observations and wide confidence interval. |
| § Any psychological financial hardship was defined as having responded yes to one or more of the individual psychological financial hardship measures, including ever being worried about paying large medical bills, worried about family's financial stability, and/or concerned about keeping job and income or earnings because of cancer. |
| ^\|\|^ Any behavioral financial hardship was defined as having responded yes to one or more of the adherence measures, including ever delaying, forgoing, or changing because of cost prescription medicine, visit to specialist, follow up care, treatment, mental health services, and/or other. Treatment and mental health services were not shown as individual measures because of the small number of observations and wide confidence intervals.  ^¶^ Any financial sacrifices were defined as having responded yes to one or more of the individual financial sacrifice measures, including ever reduce spending on vacation or leisure activities, delay large purchases, reduce basic spending, such as food and clothing, use savings set aside for other purposes, change living situation, and/or other sacrifice because of cancer. Other sacrifice was not shown as an individual measure because of the small number of observations and wide confidence interval. |

**eTable 5**. Associations of Health Insurance Literacy Problems and Financial Hardship and Financial Sacrifices among Cancer Survivors Aged ≥65 Years, by Federal Poverty Line, Medical Expenditure Panel Survey Cancer Questionnaire, 2016 (N=525)

|  | Low-income ≤138% federal poverty line | Middle-income 139-400% federal poverty line | High-income >400% federal poverty line | Wald F P ^†^ |
| --- | --- | --- | --- | --- |
|  |  |  |  |  |
|  |  |  |  |  |
|  | Adjusted OR and 95% Confidence Intervals * | Adjusted OR and 95% Confidence Intervals * | Adjusted OR and 95% Confidence Intervals * |  |
| Material financial hardship |  |  |  |  |
| Had to borrow money or go into debt | 2.77 (0.39 - 19.79) | 2.42 (0.63 - 9.30) | 1.79 (0.17 - 18.72) | 0.963 |
| Unable to cover share of the costs of medical care | 4.24 (1.19 - 15.08) | 1.77 (0.53 - 5.88) | 3.41 (0.71 - 16.31) | 0.538 |
| Any material financial hardship ^‡^ | 5.08 (1.44 - 17.83) | 2.20 (0.81 - 6.03) | 2.98 (0.75 - 11.82) | 0.545 |
| Psychological financial hardship |  |  |  |  |
| Worried about paying large medical bills | 15.54 (3.75 - 64.49) | 6.70 (2.13 - 21.12) | 7.44 (2.50 - 22.19) | 0.625 |
| Worried about family's financial stability | 18.20 (4.85 - 68.28) | 4.47 (1.59 - 12.53) | 11.77 (3.52 - 39.42) | 0.226 |
| Concerned about keeping job and income or earnings | 2.26 (0.42 - 12.05) | 3.73 (1.24 - 11.23) | 3.95 (1.10 - 14.12) | 0.859 |
| Any psychological financial hardship ^§^ | 19.23 (4.25 - 86.95) | 5.30 (1.61 - 17.48) | 8.09 (2.66 - 24.59) | 0.416 |
| Behavioral financial hardship |  |  |  |  |
| Delay or forgo cancer care because of cost -- |  |  |  |  |
| Prescription medicine | 1.44 (0.19 - 11.17) | 10.54 (1.94 - 57.34) | 2.44 (0.38 - 15.64) | 0.255 |
| Visit to specialist | 1.29 (0.12 - 13.72) | 6.17 (0.93 - 41.11) | -- | 0.141 |
| Follow up care | 5.25 (0.79 - 35.10) | 0.90 (0.25 - 3.23) | 22.68 (1.84 - 80.30) | 0.056 |
| Any behavioral financial hardship ^\|\|^ | 1.19 (0.36 - 4.00) | 1.39 (0.52 - 3.71) | 0.58 (0.17 - 1.99) | 0.525 |
| Financial sacrifice |  |  |  |  |
| Reduce spending on vacation or leisure activities | 2.64 (0.70 - 10.03) | 1.54 (0.64 - 3.72) | 3.51 (1.28 - 9.65) | 0.533 |
| Delay large purchases | 4.02 (0.81 - 19.92) | 1.84 (0.61 - 5.58) | 4.27 (1.15 - 15.80) | 0.596 |
| Basic spending | 1.88 (0.53 - 6.60) | 2.44 (1.00 - 6.01) | 4.10 (1.03 - 16.34) | 0.702 |
| Savings set aside for other purposes | 3.68 (1.06 - 12.73) | 3.73 (1.27 - 10.94) | 8.21 (2.59 - 26.00) | 0.516 |
| Living situation | 7.15 (1.10 - 46.33) | 1.88 (0.62 - 5.68) | 5.52 (1.34 - 22.72) | 0.387 |
| Any financial sacrifices ^¶^ | 1.53 (0.53 - 4.46) | 2.18 (0.78 - 6.08) | 2.31 (0.83 - 6.37) | 0.836 |

| * OR, odds ratio. Reference group was cancer survivors had no problems understanding health insurance or medical bills. Multivariable models adjusted for age group, sex, race/ethnicity, current marital status, educational attainment, current health insurance coverage, years since last cancer treatment, and number of conditions. |
| --- |
| ^†^ Wald F *P* tested the differences in the associations between health insurance literacy and financial hardship across current health insurance coverage type. |
| ^‡^Any material financial hardship was defined as having responded yes to one or more of the individual material financial hardship measures, including ever having to borrow money or go into debt because of cancer, being unable to cover share of the cost of medical care visits for cancer, and/or file for bankruptcy because of cancer. File for bankruptcy because of cancer was not shown as an individual measure because of the small number of observations and wide confidence interval. |
| ^‡^ Any psychological financial hardship was defined as having responded yes to one or more of the individual psychological financial hardship measures, including ever being worried about paying large medical bills, worried about family's financial stability, and/or concerned about keeping job and income or earnings because of cancer. |
| ^\|\|^ Any behavioral financial hardship was defined as having responded yes to one or more of the adherence measures, including ever delaying, forgoing, or changing because of cost prescription medicine, visit to specialist, follow up care, treatment, mental health services, and/or other. Treatment and mental health services were not shown as individual measures because of the small number of observations and wide confidence intervals.  ^¶^ Any financial sacrifices were defined as having responded yes to one or more of the individual financial sacrifice measures, including ever reduce spending on vacation or leisure activities, delay large purchases, reduce basic spending, such as food and clothing, use savings set aside for other purposes, change living situation, and/or other sacrifice because of cancer. Other sacrifice was not shown as an individual measure because of the small number of observations and wide confidence interval. |

**eTable 6**. Associations of Health Insurance Literacy Problems and Financial Hardship and Financial Sacrifices among Cancer Survivors Aged 18-64 Years, by Years Since Last Cancer Treatment, Medical Expenditure Panel Survey Cancer Questionnaire, 2016 (N=389)

|  | Years since last cancer treatment: <5 | Years since last cancer treatment: ≥5 or never treated / missing | Wald F P ^†^ |
| --- | --- | --- | --- |
|  |  |  |  |
|  |  |  |  |
|  | Adjusted OR and 95% Confidence Intervals * | Adjusted OR and 95% Confidence Intervals * |  |
| Material financial hardship |  |  |  |
| Had to borrow money or go into debt | 3.10 (1.15 - 8.37) | 5.37 (1.95 - 14.82) | 0.489 |
| Unable to cover share of the costs of medical care | 1.66 (0.57 - 4.79) | 2.20 (0.74 - 6.58) | 0.730 |
| Any material financial hardship ^‡^ | 2.02 (0.75 - 5.46) | 5.67 (2.49 - 12.90) | 0.120 |
| Psychological financial hardship |  |  |  |
| Worried about paying large medical bills | 4.54 (1.88 - 10.92) | 4.89 (1.73 - 13.85) | 0.914 |
| Worried about family's financial stability | 4.24 (1.76 - 10.23) | 1.59 (3.35 - 40.10) | 0.186 |
| Concerned about keeping job and income or earnings | 2.66 (1.15 - 6.13) | 8.75 (3.54 - 21.62) | 0.059 |
| Any psychological financial hardship ^§^ | 3.31 (1.21 - 9.06) | 12.38 (4.29 - 35.72) | 0.080 |
| Behavioral financial hardship |  |  |  |
| Delay or forgo cancer care because of cost -- |  |  |  |
| Prescription medicine | 4.55 (1.65 - 12.54) | 2.57 (0.44 - 14.93) | 0.580 |
| Visit to specialist | 2.29 (0.72 - 7.23) | 2.55 (0.50 - 13.14) | 0.916 |
| Follow up care | 1.18 (0.35 - 4.03) | 2.97 (0.80 - 11.06) | 0.348 |
| Any behavioral financial hardship ^\|\|^ | 1.63 (0.64 - 4.20) | 3.14 (0.90 - 10.93) | 0.410 |
| Financial sacrifice |  |  |  |
| Reduce spending on vacation or leisure activities | 4.01 (1.58 - 10.22) | 2.78 (1.05 - 7.35) | 0.594 |
| Delay large purchases | 2.94 (1.20 - 7.20) | 5.72 (1.73 - 18.91) | 0.382 |
| Basic spending | 3.49 (1.26 - 9.69) | 6.14 (2.58 - 14.62) | 0.410 |
| Savings set aside for other purposes | 7.19 (2.80 - 18.41) | 8.05 (2.60 - 24.88) | 0.872 |
| Living situation | 3.24 (1.09 - 9.68) | 2.35 (0.69 - 7.94) | 0.687 |
| Any financial sacrifices ^¶^ | 6.22 (2.17 - 17.84) | -- | -- |

| * OR, odds ratio. Reference group was cancer survivors had no problems understanding health insurance or medical bills. Multivariable models adjusted for age group, sex, race/ethnicity, current marital status, educational attainment, current family income, current health insurance coverage, and number of conditions. |
| --- |
| ^†^ Wald F *P* tested the differences in the associations between health insurance literacy and financial hardship across current health insurance coverage type. |
| ^‡^Any material financial hardship was defined as having responded yes to one or more of the individual material financial hardship measures, including ever having to borrow money or go into debt because of cancer, being unable to cover share of the cost of medical care visits for cancer, and/or file for bankruptcy because of cancer. File for bankruptcy because of cancer was not shown as an individual measure because of the small number of observations and wide confidence interval. |
| ^§^ Any psychological financial hardship was defined as having responded yes to one or more of the individual psychological financial hardship measures, including ever being worried about paying large medical bills, worried about family's financial stability, and/or concerned about keeping job and income or earnings because of cancer. |
| ^\|\|^ Any behavioral financial hardship was defined as having responded yes to one or more of the adherence measures, including ever delaying, forgoing, or changing because of cost prescription medicine, visit to specialist, follow up care, treatment, mental health services, and/or other. Treatment and mental health services were not shown as individual measures because of the small number of observations and wide confidence intervals.  ^¶^ Any financial sacrifices were defined as having responded yes to one or more of the individual financial sacrifice measures, including ever reduce spending on vacation or leisure activities, delay large purchases, reduce basic spending, such as food and clothing, use savings set aside for other purposes, change living situation, and/or other sacrifice because of cancer. Other sacrifice was not shown as an individual measure because of the small number of observations and wide confidence interval. |

**eTable 7**. Associations of Health Insurance Literacy Problems and Financial Hardship and Financial Sacrifices among Cancer Survivors Aged ≥65 Years, by Years Since Last Cancer Treatment, Medical Expenditure Panel Survey Cancer Questionnaire, 2016 (N=525)

|  | Years since last cancer treatment: <5 | Years since last cancer treatment: ≥5 or never treated / missing | Wald F P ^†^ |
| --- | --- | --- | --- |
|  |  |  |  |
|  |  |  |  |
|  | Adjusted OR and 95% Confidence Intervals * | Adjusted OR and 95% Confidence Intervals * |  |
| Material financial hardship |  |  |  |
| Had to borrow money or go into debt | 0.97 (0.27 - 3.48) | 6.42 (1.59 - 25.87) | 0.049 |
| Unable to cover share of the costs of medical care | 1.75 (0.59 - 5.19) | 4.69 (1.79 - 12.28) | 0.161 |
| Any material financial hardship ^‡^ | 1.50 (0.57 - 3.91) | 6.77 (2.75 - 16.69) | 0.018 |
| Psychological financial hardship |  |  |  |
| Worried about paying large medical bills | 3.76 (1.69 - 8.36) | 6.76 (6.79 - 41.36) | 0.015 |
| Worried about family's financial stability | 3.90 (1.64 - 9.30) | 7.13 (6.58 - 44.58) | 0.032 |
| Concerned about keeping job and income or earnings | 0.68 (0.22 - 2.06) | 9.36 (3.65 - 24.06) | 0.001 |
| Any psychological financial hardship ^§^ | 3.17 (1.41 - 7.10) | 8.77 (7.30 - 48.27) | 0.007 |
| Behavioral financial hardship |  |  |  |
| Delay or forgo cancer care because of cost -- |  |  |  |
| Prescription medicine | 2.98 (0.62 - 14.34) | 3.81 (1.05 - 13.92) | 0.817 |
| Visit to specialist | 3.16 (0.51 - 19.51) | 4.19 (0.50 - 35.07) | 0.844 |
| Follow up care | 1.98 (0.59 - 6.67) | 2.83 (0.58 - 13.88) | 0.714 |
| Any behavioral financial hardship ^\|\|^ | 0.83 (0.34 - 2.01) | 1.31 (0.55 - 3.14) | 0.045 |
| Financial sacrifice |  |  |  |
| Reduce spending on vacation or leisure activities | 3.09 (1.43 - 6.69) | 1.76 (0.73 - 4.23) | 0.384 |
| Delay large purchases | 1.99 (0.74 - 5.31) | 4.09 (1.45 - 11.54) | 0.337 |
| Basic spending | 2.75 (1.25 - 6.05) | 2.59 (0.85 - 7.88) | 0.930 |
| Savings set aside for other purposes | 3.26 (1.19 - 8.94) | 6.28 (2.16 - 18.25) | 0.405 |
| Living situation | 4.70 (1.43 - 15.44) | 2.85 (0.90 - 9.06) | 0.558 |
| Any financial sacrifices ^¶^ | 1.47 (0.64 - 3.36) | 2.59 (1.21 - 5.53) | 0.331 |

| * OR, odds ratio. Reference group was cancer survivors had no problems understanding health insurance or medical bills. Multivariable models adjusted for age group, sex, race/ethnicity, current marital status, educational attainment, current family income, current health insurance coverage, and number of conditions |
| --- |
| ^†^ Wald F *P* tested the differences in the associations between health insurance literacy and financial hardship across current health insurance coverage type. |
| ^‡^Any material financial hardship was defined as having responded yes to one or more of the individual material financial hardship measures, including ever having to borrow money or go into debt because of cancer, being unable to cover share of the cost of medical care visits for cancer, and/or file for bankruptcy because of cancer. File for bankruptcy because of cancer was not shown as an individual measure because of the small number of observations and wide confidence interval. |
| ^§^ Any psychological financial hardship was defined as having responded yes to one or more of the individual psychological financial hardship measures, including ever being worried about paying large medical bills, worried about family's financial stability, and/or concerned about keeping job and income or earnings because of cancer. |
| ^\|\|^ Any behavioral financial hardship was defined as having responded yes to one or more of the adherence measures, including ever delaying, forgoing, or changing because of cost prescription medicine, visit to specialist, follow up care, treatment, mental health services, and/or other. Treatment and mental health services were not shown as individual measures because of the small number of observations and wide confidence intervals.  ^¶^ Any financial sacrifices were defined as having responded yes to one or more of the individual financial sacrifice measures, including ever reduce spending on vacation or leisure activities, delay large purchases, reduce basic spending, such as food and clothing, use savings set aside for other purposes, change living situation, and/or other sacrifice because of cancer. Other sacrifice was not shown as an individual measure because of the small number of observations and wide confidence interval. |

**eTable 8**. Associations of Health Insurance Literacy Problems and Financial Hardship and Financial Sacrifices among Cancer Survivors Aged 18-64 Years, by Number of Current Conditions (Excluding Cancer), Medical Expenditure Panel Survey Cancer Questionnaire, 2016 (N=389)

|  | Number of current conditions (excluding cancer): 0-1 | Number of current conditions (excluding cancer): 2-8 | Wald F P ^†^ |
| --- | --- | --- | --- |
|  |  |  |  |
|  |  |  |  |
|  | Adjusted OR and 95% Confidence Intervals * | Adjusted OR and 95% Confidence Intervals * |  |
| Material financial hardship |  |  |  |
| Had to borrow money or go into debt | 2.79 (0.94 - 8.24) | 5.08 (1.97 - 13.07) | 0.453 |
| Unable to cover share of the costs of medical care | 2.17 (0.65 - 7.21) | 1.70 (0.69 - 4.21) | 0.752 |
| Any material financial hardship ^‡^ | 2.85 (0.82 - 9.93) | 3.25 (1.45 - 7.29) | 0.863 |
| Psychological financial hardship |  |  |  |
| Worried about paying large medical bills | 2.69 (0.99 - 7.26) | 7.11 (2.76 - 18.28) | 0.170 |
| Worried about family's financial stability | 3.91 (1.37 - 11.18) | 9.35 (3.25 - 26.92) | 0.239 |
| Concerned about keeping job and income or earnings | 3.13 (1.15 - 8.50) | 5.45 (2.37 - 12.51) | 0.395 |
| Any psychological financial hardship ^§^ | 2.70 (0.80 - 9.10) | 9.89 (3.67 - 26.66) | 0.109 |
| Behavioral financial hardship |  |  |  |
| Delay or forgo cancer care because of cost -- |  |  |  |
| Prescription medicine | 3.28 (0.70 - 15.41) | 3.58 (1.11 - 11.57) | 0.930 |
| Visit to specialist | 1.47 (0.32 - 6.81) | 3.38 (0.92 - 12.44) | 0.406 |
| Follow up care | 1.08 (0.20 - 5.74) | 2.94 (0.98 - 8.84) | 0.320 |
| Any behavioral financial hardship ^\|\|^ | 1.49 (0.52 - 4.31) | 2.64 (0.93 - 7.49) | 0.444 |
| Financial sacrifice |  |  |  |
| Reduce spending on vacation or leisure activities | 2.49 (0.90 - 6.91) | 4.38 (1.88 - 10.18) | 0.395 |
| Delay large purchases | 4.50 (1.38 - 14.69) | 3.44 (1.38 - 8.59) | 0.729 |
| Basic spending | 3.01 (0.91 - 9.97) | 5.71 (2.34 - 13.96) | 0.403 |
| Savings set aside for other purposes | 5.76 (2.12 - 15.64) | 9.51 (3.32 - 27.21) | 0.474 |
| Living situation | 1.32 (0.40 - 4.33) | 4.71 (1.54 - 14.39) | 0.109 |
| Any financial sacrifices ^¶^ | 30.57 (5.56 - 168.15) | 6.81 (2.30 - 20.12) | 0.149 |

| * OR, odds ratio. Reference group was cancer survivors had no problems understanding health insurance or medical bills. Conditions include arthritis, asthma, diabetes, emphysema, heart disease (angina, coronary heart disease, heart attack, other heart condition/disease), high cholesterol, hypertension, and stroke. Multivariable models adjusted for age group, sex, race/ethnicity, current marital status, educational attainment, current family income, current health insurance coverage, and years since last cancer treatment. |
| --- |
| ^†^ Wald F *P* tested the differences in the associations between health insurance literacy and financial hardship across current health insurance coverage type. |
| ^‡^Any material financial hardship was defined as having responded yes to one or more of the individual material financial hardship measures, including ever having to borrow money or go into debt because of cancer, being unable to cover share of the cost of medical care visits for cancer, and/or file for bankruptcy because of cancer. File for bankruptcy because of cancer was not shown as an individual measure because of the small number of observations and wide confidence interval. |
| ^§^ Any psychological financial hardship was defined as having responded yes to one or more of the individual psychological financial hardship measures, including ever being worried about paying large medical bills, worried about family's financial stability, and/or concerned about keeping job and income or earnings because of cancer. |
| ^\|\|^ Any behavioral financial hardship was defined as having responded yes to one or more of the adherence measures, including ever delaying, forgoing, or changing because of cost prescription medicine, visit to specialist, follow up care, treatment, mental health services, and/or other. Treatment and mental health services were not shown as individual measures because of the small number of observations and wide confidence intervals.  ^¶^ Any financial sacrifices were defined as having responded yes to one or more of the individual financial sacrifice measures, including ever reduce spending on vacation or leisure activities, delay large purchases, reduce basic spending, such as food and clothing, use savings set aside for other purposes, change living situation, and/or other sacrifice because of cancer. Other sacrifice was not shown as an individual measure because of the small number of observations and wide confidence interval. |

**eTable 9**. Associations of Health Insurance Literacy Problems and Financial Hardship and Financial Sacrifices among Cancer Survivors Aged ≥65 Years, by Number of Current Conditions (Excluding Cancer), Medical Expenditure Panel Survey Cancer Questionnaire, 2016 (N=525)

|  | Number of current conditions (excluding cancer): 0-1 | Number of current conditions (excluding cancer): 2-8 | Wald F P ^†^ |
| --- | --- | --- | --- |
|  |  |  |  |
|  |  |  |  |
|  | Adjusted OR and 95% Confidence Intervals* | Adjusted OR and 95% Confidence Intervals* |  |
| Material financial hardship |  |  |  |
| Had to borrow money or go into debt | -- | 2.69 (0.99 - 7.30) | -- |
| Unable to cover share of the costs of medical care | 20.17 (1.30 - 313.06) | 2.41 (1.07 - 5.44) | 0.150 |
| Any material financial hardship ^‡^ | 5.20 (0.48 - 55.91) | 2.92 (1.41 - 6.05) | 0.645 |
| Psychological financial hardship |  |  |  |
| Worried about paying large medical bills | 5.20 (0.84 - 32.05) | 9.14 (4.45 - 18.78) | 0.586 |
| Worried about family's financial stability | 2.73 (0.55 - 13.49) | 0.20 (4.85 - 21.46) | 0.165 |
| Concerned about keeping job and income or earnings | 3.17 (0.44 - 22.63) | 3.61 (1.63 - 7.98) | 0.904 |
| Any psychological financial hardship ^§^ | 3.88 (0.65 - 23.13) | 9.48 (4.46 - 20.16) | 0.395 |
| Behavioral financial hardship |  |  |  |
| Delay or forgo cancer care because of cost -- |  |  |  |
| Prescription medicine | 9.12 (0.72 - 114.96) | 3.18 (1.14 - 8.89) | 0.431 |
| Visit to specialist |  | 3.89 (0.81 - 18.68) |  |
| Follow up care | 40.62 (2.12 - 778.34) | 1.89 (0.63 - 5.72) | 0.053 |
| Any behavioral financial hardship ^\|\|^ | 1.06 (0.18 - 6.27) | 1.07 (0.53 - 2.13) | 0.994 |
| Financial sacrifice |  |  |  |
| Reduce spending on vacation or leisure activities | 4.73 (0.97 - 22.99) | 2.08 (1.16 - 3.73) | 0.355 |
| Delay large purchases | 1.94 (0.33 - 11.41) | 2.96 (1.34 - 6.52) | 0.680 |
| Basic spending | 2.62 (0.52 - 13.15) | 2.69 (1.27 - 5.69) | 0.978 |
| Savings set aside for other purposes | 4.89 (0.65 - 37.05) | 4.47 (2.07 - 9.66) | 0.936 |
| Living situation | 13.99 (0.86 - 226.30) | 3.34 (1.43 - 7.81) | 0.344 |
| Any financial sacrifices ^¶^ | 3.76 (0.89 - 15.96) | 1.88 (1.01 - 3.48) | 0.396 |

| * OR, odds ratio. Reference group was cancer survivors had no problems understanding health insurance or medical bills. Conditions include arthritis, asthma, diabetes, emphysema, heart disease (angina, coronary heart disease, heart attack, other heart condition/disease), high cholesterol, hypertension, and stroke. Multivariable models adjusted for age group, sex, race/ethnicity, current marital status, educational attainment, current family income, current health insurance coverage, and years since last cancer treatment. |
| --- |
| ^†^ Wald F *P* tested the differences in the associations between health insurance literacy and financial hardship across current health insurance coverage type. |
| ^‡^Any material financial hardship was defined as having responded yes to one or more of the individual material financial hardship measures, including ever having to borrow money or go into debt because of cancer, being unable to cover share of the cost of medical care visits for cancer, and/or file for bankruptcy because of cancer. File for bankruptcy because of cancer was not shown as an individual measure because of the small number of observations and wide confidence interval. |
| ^§^ Any psychological financial hardship was defined as having responded yes to one or more of the individual psychological financial hardship measures, including ever being worried about paying large medical bills, worried about family's financial stability, and/or concerned about keeping job and income or earnings because of cancer. |
| ^\|\|^ Any behavioral financial hardship was defined as having responded yes to one or more of the adherence measures, including ever delaying, forgoing, or changing because of cost prescription medicine, visit to specialist, follow up care, treatment, mental health services, and/or other. Treatment and mental health services were not shown as individual measures because of the small number of observations and wide confidence intervals.  ^¶^ Any financial sacrifices were defined as having responded yes to one or more of the individual financial sacrifice measures, including ever reduce spending on vacation or leisure activities, delay large purchases, reduce basic spending, such as food and clothing, use savings set aside for other purposes, change living situation, and/or other sacrifice because of cancer. Other sacrifice was not shown as an individual measure because of the small number of observations and wide confidence interval. |

**A**

Chi-Square P=0.011

**B**

Chi-Square P=0.027

Chi-Square P=0.149

Chi-Square P=0.004

**C**

Chi-Square P=0.064

Chi-Square P=0.327

**D**

**eFigure 1**. Prevalence of health insurance literacy problems among cancer survivors, by health insurance coverage (A), family income as a percentage of federal poverty line (B), time since last cancer treatment (C), and number of conditions excluding cancer (D). Chi-Square P

Data from Medical Expenditure Panel Survey, 2016.

eMethods on data and sample

The Medical Expenditure Panel Survey (MEPS) is a nationally representative survey of

health care use, expenditures, sources of payment, and health insurance coverage for the U.S.

civilian noninstitutionalized population. The MEPS has been used as an important data source for health services research and was also selected as a key data source for Healthy People 2020 to monitor the health and health services use in the United States. Beginning in 2002, the annual sample size for the MEPS Household Component has been 13,000-15,000 families. The full-year household core response rate has generally been about 66%.

The MEPS consists of three components: (1) the Household Component (HC), a nationally representative survey of the civilian noninstitutionalized population; (2) the Medical Provider Component, which collects data from medical care providers and facilities reported as providing care to persons interviewed in the Household Component; and (3) the Insurance Component, which collects data on the types and costs of workplace health insurance. This study used data from the MEPS-HC component. The MEPS-HC is a complex national probability survey of the U.S. civilian noninstitutionalized population. Each year a new panel of households is representatively selected from among those households that initially characterized as responding to the previous year’s National Health Interview Survey (NHIS), another large ongoing Federal health survey conducted by the National Center for Health Statistics of the Centers for Disease Control and Prevention. The NHIS is a complex multi-stage sample design. The first stage of sample selection is an area sample of primary survey units (PSUs), generally consisting of one or more counties. The second stage is to form density strata within each PSU, generally reflecting the density of minority populations for single or groups of blocks or block equivalents that are assigned to the strata. The third stage is to form “supersegments” within each stratum, consisting of clusters of housing units. Finally, households within supersegments are selected for each calendar. These features of the NHIS complex survey design carry over to the MEPS. For each new MEPS annual sample, data are collected through a series of five rounds of computer-assisted personal interviews over 30 months to yield annual data for two full calendar years.

The MEPS Experiences with Cancer Survey was sponsored and developed by the Agency for Healthcare Research and Quality, the National Cancer Institute, the American Cancer Society, the Centers for Disease Control and Prevention, the NIH Office of Behavioral and Social Sciences Research, and LIVESTRONG to improve the quality of data for estimating the cancer survivorship burden in the United States. The MEPS Cancer Self-Administered Questionnaire (CSAQ), a paper-and-pencil questionnaire, was fielded during Panel 20 Round 3 and Panel 21 Round 1 for adults who confirmed a cancer diagnosis based on the question in the MEPS-HC: (Have/Has) (PERSON) ever been told by a doctor or other health professional that (PERSON) had cancer or a malignancy of any kind? A weight variable (CSAQW16F) is to be used with the CSAQ for persons who were age 18 and older at the interview date. This weight adjusts for CSAQ nonresponse and is an estimate of the adult population self-reporting as having been diagnosed with or treated for cancer as an adult.

Adult cancer survivors were defined as those aged ≥18 years reported ever being told by a doctor or other health professional that they had cancer or a malignancy of any kind. Individuals diagnosed only with nonmelanoma skin cancer and/or skin cancer with unknown kind (n=267), aged ≥ 65 years without Medicare coverage (n=6), or who did not respond to the health insurance and financial literacy question (n=49) were excluded from this study.
